# Kontaktallergie auf ein elektronisches Gerät

**DOI:** 10.1111/ddg.15775_g

**Published:** 2025-09-15

**Authors:** Margitta Worm, Julia Oberschmied, Elsbeth Oestmann

**Affiliations:** ^1^ Klinik für Dermatologie Venerologie und Allergologie Charité – Universitätsmedizin Berlin

Sehr geehrte Herausgeber,

Apple‐Produkte werden weltweit häufig verwendet, darunter Kopfhörer, Smartphones, Uhren und andere elektronische Geräte. Bei Kopfhörern und Uhren besteht durch den direkten Hautkontakt der Geräte die Gefahr einer Kontaktsensibilisierung. Obwohl diese in der Allgemeinbevölkerung häufig verwendet werden, wurden nur wenige Fälle von allergischer Kontaktdermatitis aufgrund solcher Produkte gemeldet.^1^, ^2^, ^3^ Acrylate wurden aufgrund von Epikutantests als auslösende Kontaktallergene vermutet. Kopfhörer von Apple^®^, wie AirPods^®^, können Spuren von Acrylaten und Methacrylaten aus Klebstoffen enthalten.^4^

Wir berichten über einen 53‐jährigen männlichen Patienten, der sich mit ekzematösen Hautläsionen am linken Handrücken im Kontaktbereich seiner Apple Watch^®^ vorstellte (Abbildung 1). Er berichtete zudem über ekzematöse Hautreaktionen im äußeren Gehörgang nach dem Tragen seiner Apple AirPod^®^ In‐Ear‐Kopfhörer. Allergien waren bisher nicht bekannt, jedoch wurden ein chronischer Husten und Asthma angegeben. Der Patient hielt Katzen im Haus. Eine regelmäßige Medikamenteneinnahme wurde verneint. Nach einem zahnärztlichen Eingriff vor mehreren Jahren, bei dem Füllungsmaterial unbekannter Zusammensetzung verwendet wurde, traten Beschwerden der Mundschleimhaut auf.

**ABBILDUNG 1:**
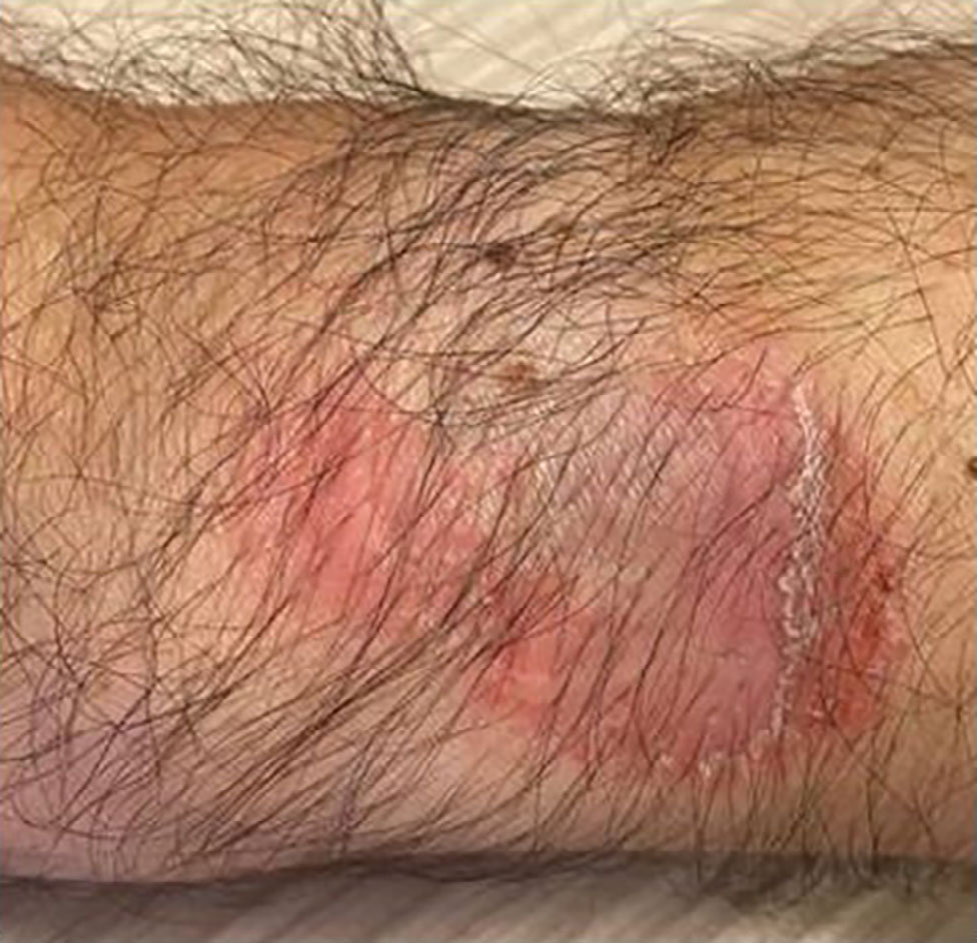
Ekzematöse Hautläsion im Kontaktbereich der Uhr.

Der Epikutantest mit der Standard‐Testreihe und der Zahntechniker‐Testreihe der Deutschen Kontaktallergie‐Gruppe (DKG) ergab zweifach positive (++) Reaktionen auf Hydroxyethylacrylat, 2‐Hydroxypropylmethacrylat, Isobornylacrylat und positive Reaktionen (+) auf 2 ‐Hydroxyethylmethacrylat, Ethylenglykoldimethacrylat, Triethylenglykoldimethacrylat nach 72 Stunden (Abbildung 2). Der Pricktest für die häufigsten Inhalationsallergene war positiv auf Hausstaubmilben. Das Gesamt‐IgE betrug 24 KU/l. Es wurde kein spezifisches IgE gegen Katzen und nur ein sehr geringes spezifisches IgE gegen Hausstaubmilben gefunden. Ein Differenzialblutbild, ein Lungenfunktionstest und der NO‐Test (Messung des ausgeatmeten Stickoxids) waren normal.

**ABBILDUNG 2:**
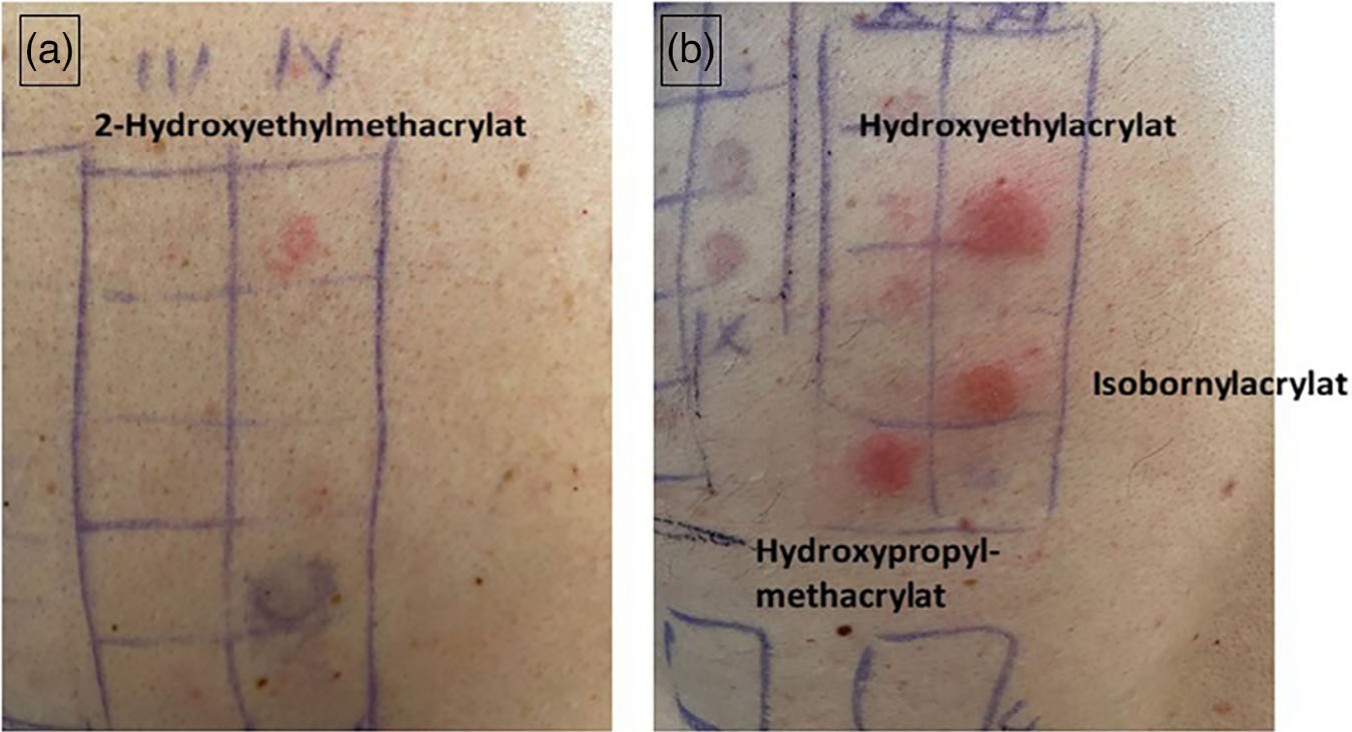
(a, b) Positive Epikutantestreaktionen gegen Acrylate.

Wir identifizierten Acrylate und Methacrylate als Ursache der ekzematösen Hautläsionen im äußeren Gehörgang und am Handgelenk aufgrund der Exposition gegenüber den Air Pods^®^ und der Apple Watch^®^. Elektronische Geräte, die ständig in engem Kontakt mit der Haut stehen, können aufgrund ihres Acrylatgehalts eine Kontaktsensibilisierung hervorrufen. Es ist bekannt, dass Acrylatmonomere starke Sensibilisatoren sind,^2^, ^4^ während das Risiko einer Allergie gegen polymerisierte Acrylate und Methacrylate gering ist. Eine unvollständige Polymerisation kann jedoch zu Spuren von Monomeren führen.^2^ Interessanterweise beobachteten wir bei unserem Patienten auch eine deutliche Reaktivität gegenüber Isobornylacrylat und 2‐Hydroxypropylacrylat. Es wurde beschrieben, dass Isobornylacrylat in Geräten zur kontinuierlichen Glukoseüberwachung bei Patienten mit Typ‐1‐Diabetes ein starker Sensibilisator ist.^5^, ^6^ Es wird vermutet, dass eine Sensibilisierung gegen Isobornylacrylat bei Glukoseüberwachungssystemen durch die Diffusion des Allergens aus dem Kleber resultiert, der bei der Montage verwendet wird.^2^ Darüber hinaus wurde Isobornylacrylat auch als berufliches Kontaktallergen in Schutzfolien von Mobiltelefonen beschrieben.^7^

Bei Patienten mit einer Kontaktallergie gegen AirPods^®^ wurde auch eine Co‐Sensibilisierung gegenüber Isobornylacrylat und 2‐Hydroxypropylacrylat in Kombination mit einer Methylacrylat‐Sensibilisierung beschrieben.^2^

Wir vermuten, dass die Sensibilisierung bei unserem Patienten während eines zahnärztlichen Eingriffs mit acrylathaltigem Füllungsmaterial aufgetreten ist. Die Exposition gegenüber unpolymerisierten (Meth)acrylaten ist eine relevante Ursache für die berufsbedingte allergische Kontaktdermatitis bei Zahntechnikern und Zahnärzten.^8^ Die klinischen Reaktionen, möglicherweise aber auch Sensibilisierungen im Zusammenhang mit dem AirPod^®^ und der Apple Watch^®^ selbst, könnten auf die intensive Exposition zurückzuführen sein. Heutzutage sind in Medizinprodukten verschiedene Acrylate und Methacrylate enthalten, unter anderem in orthopädischen Materialien, chirurgischen Klebstoffen und Klebebändern. Auch in Nagelkosmetikprodukten sind (Meth)acrylate häufig enthalten, was für Verbraucher und Fachleute ein zunehmendes Gesundheitsproblem darstellt. Sie wurden kürzlich als neu auftretende Kontaktallergene deklariert.^4^, ^9^ Insbesondere die fehlende Kennzeichnung der Inhaltsstoffe in Medizinprodukten stellt immer noch ein Hindernis für die Untersuchung allergischer Reaktionen dar. Derzeit fördern die breiten arbeits‐ und gesundheitsbezogenen Aspekte der Acrylatallergie sowie die stark steigende Inzidenz die Diskussion darüber, wie das Risiko für Verbraucher und Patienten gemindert werden kann.

## DANKSAGUNG

Open access Veröffentlichung ermöglicht und organisiert durch Projekt DEAL.

## INTERESSENKONFLIKT

Keiner.
